# The role of pulmonary function in patients with heart failure and preserved ejection fraction: Looking beyond chronic obstructive pulmonary disease

**DOI:** 10.1371/journal.pone.0235152

**Published:** 2020-07-07

**Authors:** Wei-Ming Huang, Jia-Yih Feng, Hao-Min Cheng, Su-Zhen Chen, Chi-Jung Huang, Chao-Yu Guo, Wen-Chung Yu, Chen-Huan Chen, Shih-Hsien Sung

**Affiliations:** 1 Department of Medicine, Taipei Veterans General Hospital, Taipei, Taiwan; 2 Department of Medicine, National Yang-Ming University School of Medicine, Taipei, Taiwan; 3 Department of Chest Medicine, Taipei Veterans General Hospital, Taipei, Taiwan; 4 Department of Medical Education, Taipei Veterans General Hospital, Taipei, Taiwan; 5 Department of Public Health, National Yang-Ming University School of Medicine, Taipei, Taiwan; University of Dundee, UNITED KINGDOM

## Abstract

**Background:**

The prognostic value of chronic obstructive pulmonary disease (COPD) as a comorbidity in heart failure has been well documented. However, the role of pulmonary function indices in patients with heart failure and preserved ejection fraction (HFpEF) remains to be elucidated.

**Methods:**

Subjects with HFpEF received pulmonary function tests and echocardiogram. Total lung capacity (TLC), residual volume (RV), forced expiratory flow rate between 25% and 75% of vital capacity (FEF25-75), forced expiratory volume in the 1^st^ second (FEV1), forced vital capacity (FVC), and vital capacity (VC) were measured. Echocardiographic indices, including pulmonary artery systolic pressure (PASP), the ratio of early ventricular filling flow velocity to the septal mitral annulus tissue velocity (E/e’), and left ventricular mass (LVM), were recorded. National Death Registry was linked for the identification of mortality.

**Results:**

A total of 1194 patients (72.4±13.2 years, 59% men) were enrolled. PASP, E/e’ and LVM were associated with either obstructive (RV/TLC, FEV1 and FEF25-75) or restrictive (VC and TLC) ventilatory indices. During a mean follow-up of 23.0±12.8 months, 182 patients died. Subjects with COPD had a lower survival rate than those without COPD. While VC, FVC, RV/TLC, and FEV1 were all independently associated with all-cause mortality in patients without COPD, only FEF25-75 was predictive of outcomes in those with COPD.

**Conclusions:**

The abnormalities of pulmonary function were related to the cardiac hemodynamics in patients with HFpEF. In addition, these ventilatory indices were independently associated with long-term mortality, especially in those without COPD.

## Introduction

Chronic obstructive pulmonary disease (COPD) is prevalent in chronic heart failure (HF) patients with either reduced (HFrEF) or preserved left ventricular ejection fraction (HFpEF) [1, 2]. Due to the lack of routine spirometric examinations in heart failure patients, self-reported COPD history only identifies a minority of COPD in these patients, resulting in a notably under-diagnosis of COPD in patients with HF [3]. The coexistence of COPD in HF is associated with a worse prognosis, in terms of mortality and HF hospitalizations [1, 2]. On the other hand, more than 20% of the patients with stable COPD actually had concomitant HF, and the others were at high risks of developing HF [4, 5].

The cardio-pulmonary interplay was described in a general population of 15,010 subjects, demonstrating that forced expiratory volume in the 1^st^ second (FEV1), forced vital capacity (FVC) and their ratio (FEV1/FVC) were associated with left ventricular systolic and diastolic function, and N-terminal pro-B type natriuretic peptide (NT-proBNP) levels [6]. Ries et al. further observed a significant restrictive change of lung and reduction of FEV1 when pulmonary artery wedge pressure (PAWP) was of ≥20 mmHg in patients undergoing cardiac catheterization [7]. Submucosal edema related to the decompensation of HF is proposed to cause airway obstruction [8]. Whilst lung volume was reduced as a function of disease severity in patients with HFrEF [9], heart transplantation would normalize total lung capacity (TLC), FEV1 and FVC [10, 11], suggesting a causal relationship between cardiac performance and pulmonary function.

HFpEF involves cardiovascular aging and multiple comorbidities, including COPD [12, 13]. Lung function abnormalities highly prevail in patients with HFpEF, and may deteriorate during exercise [14, 15]. However, the pathophysiology and clinical relevance between heart and lung functions in HFpEF patients, regardless of the presence of COPD, remain to be elucidated. Therefore, we conducted the present study to evaluate the cardiopulmonary correlations and the prognostic impacts of pulmonary function parameters in patients with HFpEF.

## Methods

### Study population

The study population was drawn from an administrative registry to **in**vestigate **H**eart **a**nd **L**ung int**er**action (INHALER registry). The registry from August 2005 to December 2012 was composed of 8963 ambulatory outpatients who complained of exertional dyspnea. All of them have received both pulmonary function tests and echocardiographic studies. A total of 1587 subjects were diagnosed to have heart failure, based on a history of HF hospitalization or the Framingham Heart Failure Diagnostic Criteria [16], whereas 2289 subjects had COPD, diagnosed by a pulmonologist according to typical symptoms and a pre-bronchodilator FEV1/FVC ratio <0.7. Subjects with heart failure and LVEF ≥50% were defined to have HFpEF. Patients with severe hepatic disease, hematopoietic diseases, active malignancy, or asthma were excluded from this analysis. The investigation was conformed to the principles outlined in the Declaration of Helsinki. The institutional review committee of Taipei Veterans General Hospital approved the use of the registry data for research purposes, and the informed consent was waived.

Data of demographic characteristics, hemogram, biochemistry, and echocardiography were prospectively input in a web-based medical recording system. Estimated glomerular filtration (eGFR) rate was calculated by the Chinese Modification of Diet in Renal Disease equation (cMDRD) [17]. In addition, the prescribed medications were also retrieved from the system. Renin-angiotensin system blockers were referred to either angiotensin converting enzyme inhibitors or angiotensin II receptor blockers. Used bronchodilators, including oral theophylline, inhaled long-acting beta agonists (LABA), long-acting muscarinic antagonists (LAMA), and steroid, were also recorded.

The left ventricular ejection fraction (LVEF) was derived from the 2D-guided M-mode echocardiography with Teichholz method [18]. Left ventricular end-diastolic dimension (LVEDD), end-systolic dimension (LVESD), left ventricular end-diastolic volume (LVEDV), left ventricular mass (LVM), and left atrial (LA) dimension were obtained. E/A ratio represented the ratio of left ventricular early (E) to late (A) filling flow velocity. E/e’ was the ratio of early ventricular filling flow velocity (E) to the septal mitral annulus tissue velocity (e’), and an E/e’ of >15 indicated high left ventricular end-diastolic pressure (LVEDP). Pulmonary artery systolic pressure (PASP) was also estimated, and a PASP of >35mmHg was referred to pulmonary hypertension.

Pulmonary function test was performed by standard spirometry (CPFS/D USB, Medical Graphics, St Paul, Minnesota, USA) in all patients and body plethysmograph (MasterScreen Body Plethysmograph, Erich Jaeger GmbH, Würzburg, Germany) in 818 subjects. According to the statement of American Thoracic Society standards, residual volume (RV), TLC, FEV1 and FVC were presented as the percentage of their predicted values [19]. The forced expiratory flow between 25% and 75% of vital capacity (FEF25-75) was calculated. The severity of obstructive ventilation defect was graded according to the predicted %FEV1 (mild >80%; moderate 50–80%; severe 30–50%; or very severe < 30%). The severity of the restrictive ventilation defect was graded according to the predicted %TLC (normal range > 80%; mild 70–80%; moderate 50–70%; severe/very severe <50%).

The ventilatory abnormalities were further categorized into 4 types: obstructive type indicated by FEV1/FVC<70% and predicted FVC%≥80; restrictive type indicated by FEV1/FVC≥70% and predicted FVC%<80; mixed type indicated by FEV1/FVC<70% and predicted FVC%<80; and normal [19].

### Follow-up

The study population was followed for up to 3 years. The causes and dates of death were identified from the National Death Registry [20].

### Statistical analysis

Baseline characteristics were compared by Chi-square tests and Student's t-test as appropriate. Normally distributed continuous variables were presented as mean ± standard deviation. Categorical variables were reported as the absolute numbers and relative frequencies. Cox proportional hazards models were used to evaluate the independence of pulmonary function indices in the prediction of mortality with adjustments for age, sex, hemoglobin, eGFR, and PASP. Forward stepwise multiple Cox regression analyses were used to compare the predictive values between pulmonary function indices, after accounting for age, sex, hemoglobin, eGFR, and PASP. The correlates of pulmonary function parameters were examined by the linear regression analyses, and their determinants were evaluated by the stepwise multiple linear regression analyses. The attributable proportions were then calculated. All the statistics were performed using SPSS v.20.0 software (SPSS, Inc., Chicago, IL, USA). All the tests performed were two-sided and a P value of <0.05 was considered statistically significant.

## Results

**Fig 1:**
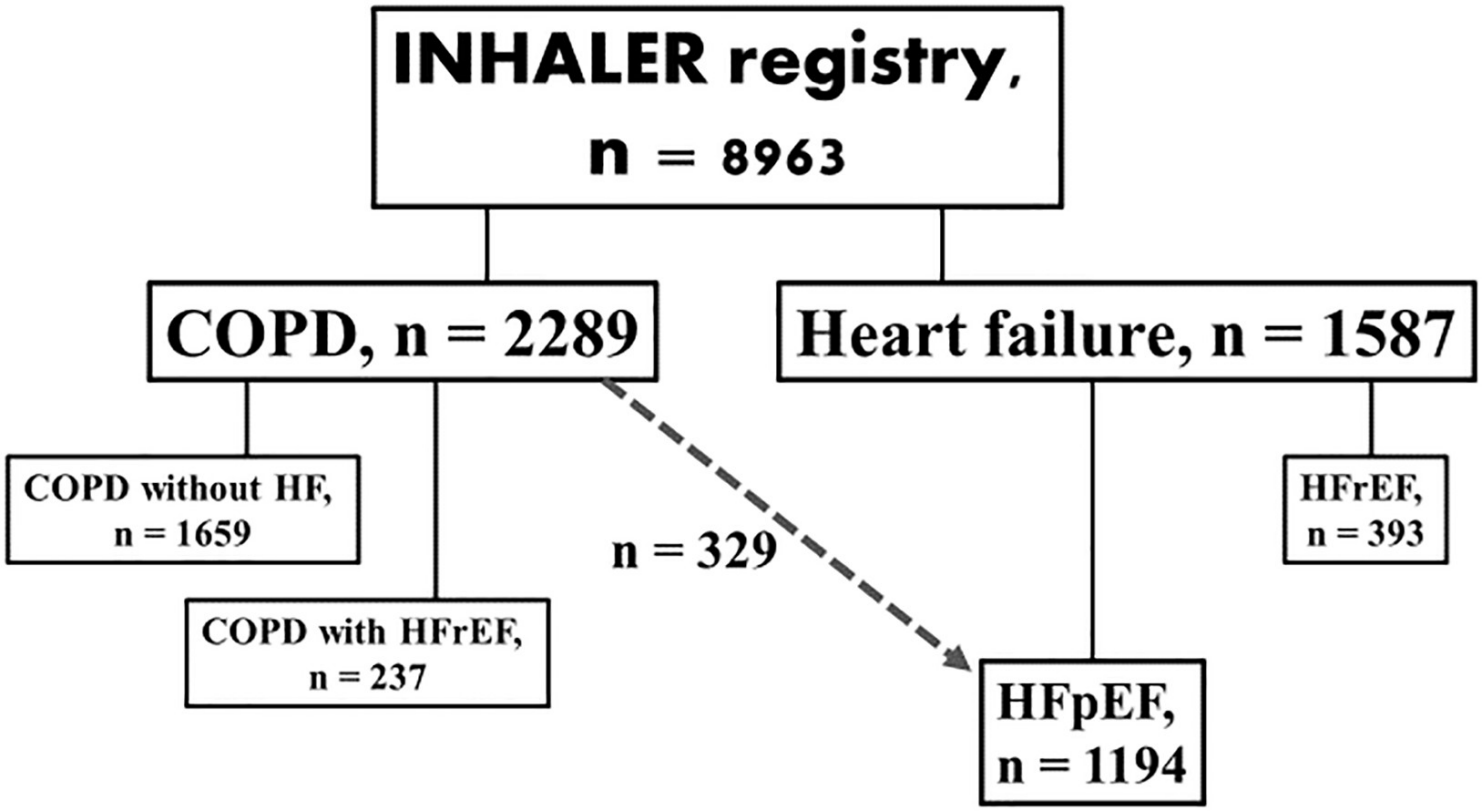
The flow chart of the study population.

A total of 1194 patients (age 72.4±13.2 years, 59% men) with HFpEF were enrolled in this analysis (Fig 1), of whom 329 subjects (27.6%) had COPD. Table 1 disclosed the comparison of baseline characteristics between the patients with and without COPD. In short, patients with COPD were older, more likely to be men and had atrial fibrillation. The prevalence of hypertension, diabetes, coronary artery disease, and stroke were similar in both groups. Patients with COPD had lower LVEF and larger LA dimension, but LVM, LVEDD, LVEDV, stroke volume (SV), PASP and E/e’ were not different compared to those without COPD. As for the pulmonary function indices, the predicted %TLC, predicted %RV and RV/TLC ratio were higher in subjects with COPD, whereas the FEF25-75, predicted %FEV1, and FEV1/FVC ratio were lower in subjects with COPD compared to those without. Both the predicted %VC and %FVC were similar in both groups.

**Table 1:** Baseline characteristics of the study population.

	HFpEF without COPD (n = 865)	HFpEF with COPD (n = 329)	P value
***Age*, *years***	70.1 ± 13.8	76.7 ± 9.9	<0.001
***Male gender*, *n (%)***	451 (52.4)	248 (75.4)	<0.001
***Co-morbidity*, *n (%)***			
Hypertension	410 (47.9)	166 (50.4)	0.345
Diabetes mellitus	207 (23.9)	66 (20.1)	0.155
Coronary artery disease	359 (41.5)	147 (44.7)	0.321
Atrial fibrillation	90 (10.4)	49 (14.9)	0.031
Stroke	64 (7.4)	28 (8.5)	0.245
***Echocardiography***			
LVEF, %	70.9 ± 9.6	69.2 ± 9.7	0.007
LV mass, gm	195.3 ± 81.1	197.6 ± 82.1	0.671
LVEDV, ml	116.4 ± 45.9	120.2 ± 51.9	0.230
LVEDD, mm	48.8 ± 8.1	49.3 ± 9.1	0.387
SV, ml	79.9 ± 31.3	81.6 ± 33.9	0.353
LA diameter, mm	42.0 ± 9.5	43.9 ± 10.9	0.004
E/A ratio	0.96 ± 0.47	0.86 ± 0.41	0.014
Septal E/E’	13.4 ± 6.9	13.0 ± 6.1	0.743
PASP, mmHg	40.1 ± 17.7	41.8 ± 19.4	0.181
***Pulmonary function test***			
Predicted RV, %	103.1 ± 31.4	110.2 ± 36.1	0.005
Predicted TLC, %	86.2 ± 18.3	89.9 ± 18.2	0.007
Predicted VC, %	75.1 ± 20.7	76.5 ± 19.8	0.364
RV/TLC ratio, %	46.1 ± 11.8	48.6 ± 10.8	0.005
FEF 25 to 75%, L/s	1.75 ± 0.86	0.65 ± 0.33	<0.001
Predicted FEV1, %	80.2 ± 23.0	65.1 ± 24.0	<0.001
Predicted FVC, %	73.1 ± 22.0	75.8 ± 23.1	0.064
FEV1/FVC ratio, %	81.4 ± 7.2	59.4 ± 9.4	<0.001
***Hemogram and Biochemistry***			
Hemoglobin, g/dl	12.1 ± 2.1	12.0 ± 1.8	0.815
eGFR, ml/min/1.73 m^2^	72.9 ± 29.8	68.7 ± 27.6	0.066
Sodium, mEq/L	138.9 ± 3.8	139.1 ± 3.5	0.523
Potassium, mEq/L	4.17 ± 0.57	4.14 ± 0.57	0.493
***Baseline Medications*, *n (%)***			
β-blockers	244 (28.2)	77 (23.4)	0.094
RAS blockers	401 (46.4)	167 (50.8)	0.174
Mineralocorticoid antagonist	172 (19.9)	73 (22.2)	0.378
Loop diuretics	326 (37.7)	147 (44.7)	0.027
***Bronchodilators*, *n (%)***			
Monotherapy, theophylline	76 (8.8)	49 (14.9)	0.002
Monotherapy, LABA	5 (0.6)	6 (1.8)	0.053
Monotherapy, LAMA	12 (1.4)	53 (16.1)	<0.001
Combination of bronchodilators	24 (2.8)	44 (13.4)	<0.001
Steroid plus either bronchodilator	32 (3.7)	51 (15.5)	<0.001

COPD: chronic obstructive pulmonary disease; E/A ratio: ratio of the early (E) to late (A) ventricular filling velocities; E/E': ratio of early ventricular filling velocity (E) to early diastolic tissue velocity mitral annulus; eGFR: estimated glomerular filtration; FEF 25 to 75%: forced expiratory flow at 25–75% of the pulmonary volume; FEV1: forced expiratory volume in 1^st^ second; FVC: forced vital capacity, HFpEF: heart failure with preserved ejection fraction; β-blockers: heart failure specific β-blockers, including bisoprolol, carvedilol, and metoprolol; LA diameter: the diameter of left atrium; LABA: long-acting beta-adrenoceptor agonist; LAMA: long-acting muscarinic antagonists; LVEDD: left ventricular end-diastolic dimension; LVEDV: left ventricular end-diastolic volume; LVEF: left ventricular ejection fraction; LV mass: left ventricular mass; PASP: pulmonary artery systolic pressure; RAS blockers: renin-angioten system blockers, including angiotensin converting enzyme inhibitors and angiotensin receptor blockers; RV: residual volume; SV: stroke volume; TLC: total lung capacity; VC: vital capacity.

The prescribed medications, including β-blockers, RAS blockers and mineralocorticoid antagonists, were similar in both groups. However, patients with COPD were more likely to be prescribed with diuretics and bronchodilators.

### The abnormalities of pulmonary function in HFpEF

**Fig 2:**
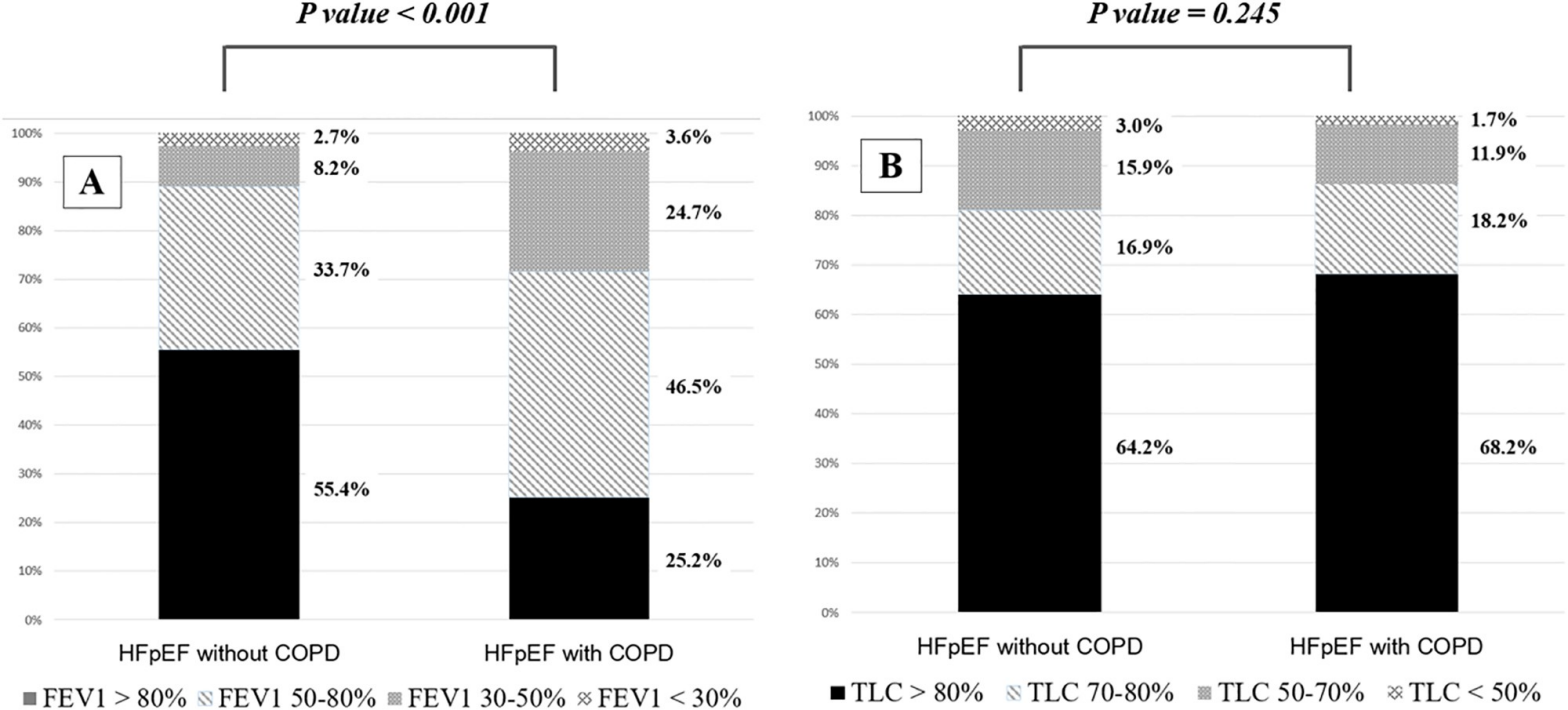
The distributions of the pulmonary function abnormalities. The severity of obstructive ventilation defect was graded according to the predicted %FEV1 (mild >80%; moderate 50–80%; severe 30–50%; or very severe < 30%). The severity of the restrictive ventilation defect was graded according to the predicted %TLC (normal range > 80%; mild 70–80%; moderate 50–70%; severe/very severe <50%).

The distribution of the pulmonary function abnormalities was shown in Fig 2. Compared to patients without COPD, obstructive ventilatory defects were more prevalent in patients with COPD that 74.8% of them had more than mild obstructive defect (p < 0.001) [21]. Even in patients without COPD, 44.6% of the patients with HFpEF patients had predicted %FEV1 of <80%.

In contrast, the distributions of restrictive ventilatory abnormalities were similar in both patients with and without COPD. 31.8% and 35.8% of the HFpEF patients with and without COPD had restrictive ventilatory pattern, respectively.

### Predictors of mortality in subjects with HFpEF

**Fig 3:**
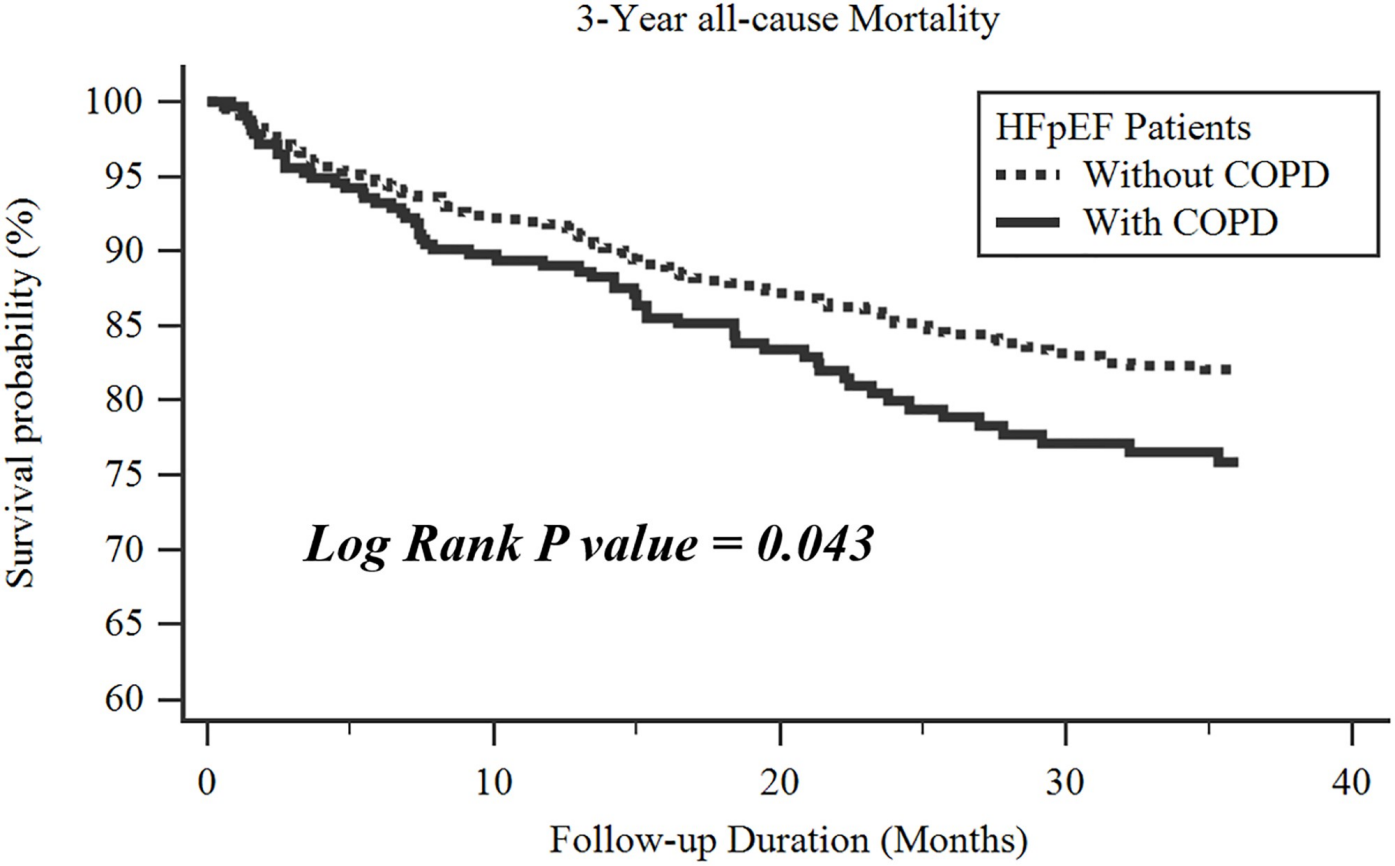
The Kaplan–Meier survival curve analysis of the study population, stratified by the presence of chronic obstructive pulmonary disease.

There were 182 deaths during a mean follow-up duration of 23.0 ± 12.8 months. Patients with COPD had a lower survival rate compared to those without. (log rank p = 0.043) (Fig 3) Among the whole study population or subjects without COPD, age, hemoglobin, eGFR, PASP, predicted %TLC, predicted %VC, RV/TLC ratio, FEF25-75, predicted %FEV1, predicted %FVC, and FEV1/FVC ratio were all related to long-term survival. (Table 2) After accounting for age, gender, hemoglobin, eGFR and PASP, the predicted %VC, RV/TLC ratio, predicted %FEV1 and predicted %FVC remained associated with mortality. (Table 3) In a forward stepwise Cox regression analysis among the pulmonary function indices, predicted %VC was the strongest predictor getting into the model in the whole study population [hazard ratios and 95% confidence interval: 0.984 (0.973–0.994) and in subjects without COPD [0.978 (0.965–0.992)].

**Table 2:** Predictors of 3-year mortality identified by univariate Cox regression analysis.

	Total population		HFpEF without COPD		HFpEF with COPD	
	HR (95% CI)	P value	HR (95% CI)	P value	HR (95% CI)	P value
**Age, years**	1.025 (1.012–1.039)	<0.001	1.026 (1.011–1.042)	0.001	1.014 (0.986–1.043)	0.334
**Gender (Male)**	1.093 (0.810–1.474)	0.562	1.040 (0.727–1.487)	0.830	0.981 (0.540–1.782)	0.981
**Hemoglobin (g/dl)**	0.875 (0.813–0.943)	<0.001	0.903 (0.828–0.986)	0.022	0.797 (0.686–0.927)	0.003
**eGFR (ml/min/1.73 m^2^)**	0.988 (0.983–0.994)	<0.001	0.990 (0.984–0.996)	0.002	0.984 (0.974–0.995)	0.003
**Septal E/E’**	1.021 (0.999–1.043)	0.063	1.018 (0.991–1.046)	0.189	1.035 (0.992–1.079)	0.111
**PASP (mmHg)**	1.020 (1.014–1.026)	<0.001	1.021 (1.012–1.030)	<0.001	1.018 (1.009–1.027)	<0.001
**Predicted RV%**	1.000 (0.994–1.005)	0.882	1.000 (0.993–1.007)	0.954	0.998 (0.990–1.007)	0.715
**Predicted TLC%**	0.984 (0.975–0.993)	0.001	0.982 (0.971–0.994)	0.003	0.985 (0.972–0.999)	0.041
**Predicted VC%**	0.975 (0.966–0.983)	<0.001	0.970 (0.959–0.981)	<0.001	0.982 (0.968–0.996)	0.012
**RV/TLC ratio, %**	1.034 (1.018–1.050)	<0.001	1.038 (1.019–1.057)	<0.001	1.024 (0.996–1.052)	0.098
**FEF 25–75%, L/s**	0.567 (0.461–0.697)	<0.001	0.553 (0.427–0.716)	<0.001	0.179 (0.070–0.455)	<0.001
**Predicted FEV1%**	0.976 (0.970–0.982)	<0.001	0.975 (0.968–0.982)	<0.001	0.977 (0.965–0.990)	<0.001
**Predicted FVC%**	0.973 (0.967–0.980)	<0.001	0.971 (0.963–0.978)	<0.001	0.977 (0.965–0.989)	<0.001
**FEV1/ FVC ratio, %**	0.992 (0.980–1.004)	0.187	1.027 (1.003–1.052)	0.028	0.976 (0.951–1.001)	0.055

eGFR: estimated glomerular filtration, FEF 25 to 75%: forced expiratory flow at 25–75% of the pulmonary volume, FEV1: forced expiratory volume in 1st second, FVC: forced vital capacity, PASP: pulmonary artery systolic pressure, RV: residual volume, TLC: total lung capacity, VC: vital capacity

**Table 3:** Predictors of 3-year mortality identified by *multivariate Cox regression analysis.

	Total population		HFpEF without COPD		HFpEF with COPD	
	HR (95% CI)	P value	HR (95% CI)	P value	HR (95% CI)	P value
**Predicted TLC%**	0.996 (0.984–1.009)	0.548	0.997 (0.981–1.014)	0.720	0.994 (0.976–1.013)	0.557
**Predicted VC%**	0.984 (0.973–0.994)	0.003	0.978 (0.965–0.992)	0.002	0.990 (0.973–1.008)	0.259
**RV/TLC ratio, %**	1.027 (1.006–1.049)	0.012	1.032 (1.005–1.059)	0.019	1.024 (0.989–1.031)	0.179
**FEF 25–75%, L/s**	0.857 (0.661–1.110)	0.243	0.852 (0.615–1.181)	0.337	0.281 (0.082–0.965)	0.044
**Predicted FEV1%**	0.989 (0.981–0.997)	0.006	0.986 (0.977–0.996)	0.007	0.988 (0.972–1.005)	0.160
**Predicted FVC%**	0.986 (0.978–0.995)	0.002	0.983 (0.973–0.994)	0.002	0.989 (0.973–1.005)	0.167
**FEV1/ FVC ratio, %**	1.004 (0.990–1.019)	0.576	0.981 (0.949–1.014)	0.258	0.976 (0.951–1.001)	0.055

* after accounting for age, gender, hemoglobin, eGFR and PASP

eGFR: estimated glomerular filtration, FEF 25 to 75%: forced expiratory flow at 25–75% of the pulmonary volume, FEV1: forced expiratory volume in 1st second, FVC: forced vital capacity, PASP: pulmonary artery systolic pressure, RV: residual volume, TLC: total lung capacity, VC: vital capacity

In subjects with COPD, hemoglobin, eGFR, PASP, predicted %TLC, predicted %VC, FEF25-75, predicted %FEV1 and predicted %FVC were correlated with 3-year mortality. (Table 2) After accounting for age, gender, hemoglobin, eGFR and PASP, FEF25-75 was the only pulmonary function index predictive of 3-year mortality. [0.281 (0.082–0.965)] (Table 3).

### The associations between cardiac performance and pulmonary function in HFpEF

PASP and E/e’ constructed the best correlation model for FEF25-75 and RV/TLC ratio. PASP and E/e’ contribute to the correlation of FEF25-75 by 91.5% and 8.5%, and RV/TLC ratio by 90.3% and 9.7%, respectively. (Table 4) In contrast, PASP and LVM contributed to the correlation of predicted %VC by 95.0% and 5.0%, and predicted %TLC by 88.7% and 11.3%, respectively. However, only PASP levels was associated with predicted %FEV1.

**Table 4:** *Multivariate linear regression analysis to determine the independent predictors of pulmonary function parameters in HFpEF.

Obstructive ventilation			Restrictive ventilation			Small airway function		
Predicetd FEV1% (R-squared = 0.080)			Predicted VC % (R-squared = 0.102)			FEF 25 to 75% (R-squared = 0.059)		
	B	P value		B	P value		B	P value
**PASP**	-0.284	<0.001	**PASP**	-0.300	<0.001	**PASP**	-0.205	<0.001
			**LV mass**	-0.081	0.030	**Septal E/E’**	-0.084	0.011
**RV to TLC ratio (R-squared = 0.052)**			**Predicted TLC% (R-squared = 0.071)**					
	**B**	**P value**		**B**	**P value**			
**PASP**	0.191	<0.001	**PASP**	-0.235	<0.001			
**Septal E/E’**	0.086	0.034	**LV mass**	-0.093	0.015			

*stepwise adjusted septal E/E’, PASP, LA diameter, and Left ventricular mass

FEF 25 to 75%: forced expiratory flow at 25–75% of the pulmonary volume, FEV1: forced expiratory volume in 1st second, FVC: forced vital capacity, LA diameter: the diameter of left atrium, LV mass: left ventricular mass, PASP: pulmonary artery systolic pressure, RV: residual volume, TLC: total lung capacity, VC: vital capacity

**Fig 4:**
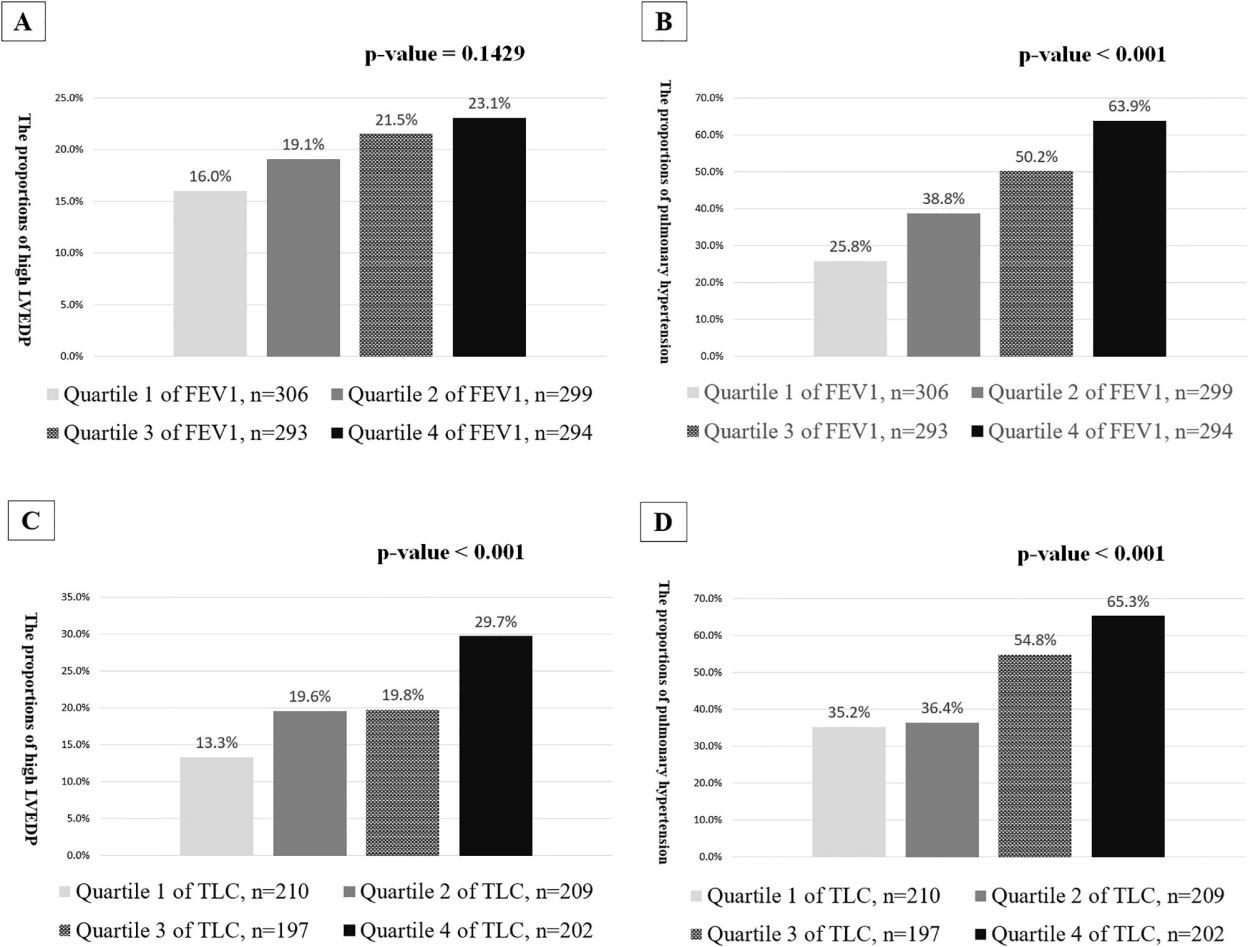
The prevalence of high left ventricular end-diastolic pressure (LVEDP) and pulmonary hypertension, according to the quartile distributions of the predicted %FEV1 (>93%, 77–93%, 60–77%, and <60%) and the predicted %TLC (>99%, 87–99%, 77–87%, and <77%).

The presence of high LVEDP were 16.0%, 19.1%, 21.5% and 23.1% along with the 4 grades of the predicted %FEV1 (p = 0.1429) and 13.3%, 19.6%, 19.8% and 29.7% along with the 4 grades of the predicted %TLC (p < 0.001) (Fig 4A and 4C). The presence of pulmonary hypertension was 25.8%, 38.8%, 50.2% and 63.9% along with the 4 grades of predicted %FEV1 (p < 0.001) and 35.2%, 36.4%, 54.8% and 65.3% along with the 4 grades of predicted %TLC (p < 0.001) (Fig 4B and 4D).

## Discussion

The present study demonstrated a high prevalence of 27.6% concomitant COPD in patients with HFpEF, who had poorer long-term survival than the others. In addition, the pulmonary function indices were independently associated with the clinical outcomes in patients with or without COPD. For subjects without COPD, both obstructive and restrictive lung impairments were related to the long-term survival. In contrast, only the small airway functional index, FEF25-75, was predictive of mortality in those with COPD. The study further showed the significant correlations between cardiac performance and pulmonary functions, while pulmonary hypertension and LVEDP were the major determinants of ventilatory abnormalities. The findings may support that pulmonary function impairment could reflect the left ventricular dysfunction and was related to poor clinical outcomes in patients with HFpEF, regardless of the presence of COPD.

### Prevalence of lung disease in HFpEF

HFpEF has been considered as a syndrome composing of heterogeneous co-morbidities, and COPD is one of the major morbidity accounting for 15% to 25% of the HF patients [1, 2]. Given that both HF and COPD share similar symptoms and signs, a standard work flow is required to make proper diagnoses [22]. While pulmonary edema might be masked in the chest radiograph in patients with COPD [23], B-type natriuretic peptide (BNP) or NT-proBNP levels could be helpful to differentiate the two diseases [24].

In previous reports, the diagnosis of COPD was made based on clinical findings and medical records rather than comprehensive pulmonary function tests [1, 2, 25, 26]. Brenner et al. suggested that pulmonary function tests were warranted to make a valid diagnosis of COPD in patients with HFrEF [27]. In this study of HFpEF, we conducted a full-scale survey of spirometry. The study clearly showed that predicted %TLC and RV/TLC ratio were higher in patients with COPD than those without COPD.

### Cardiopulmonary interaction in HFpEF

The ventilatory abnormalities in patients with HF may be resulted from the space-occupying phenomenon due to cardiomegaly [28], impaired alveolar-capillary gas exchange due to chronic lung congestion [29], and airway narrowing due to submucosal edema [8]. Baum et al. had demonstrated the significant associations between FVC and FEV1 and left ventricular end-diastolic volume, LVEDD, LVEF, stroke volume, and E/e’ in a general population of 15,010 subjects [6]. In addition, FVC, FEV1 and FEV1/FVC ratio were all predictive of the presence of HF with either reduced or preserved LVEF [6]. The improvement of pulmonary functions after cardiac resynchronization therapy or heart transplantation may support the dynamics of cardiac performance as the causes of the abnormal ventilation [10, 11, 30].

While lung function abnormalities prevail in patients with HFpEF [14], Obokata et al. had further demonstrated that ventilation reserve reduced along with the increase of PAWP and pulmonary artery pressures [31]. The present study also showed PASP as the dominant factor related to either obstructive or restrictive ventilatory impairment in patients with HFpEF. In addition, LVEDP, as indexed by E/e’ and LVM, were also associated with the pulmonary function indices. Beyond the common obstructive and restrictive ventilatory indices, this may be the first study illustrating the influence of cardiac performance on the small airway function, as indexed by FEF25-75. When patients with COPD were excluded from this analysis, PASP, E/e’ and LVM remained related to the pulmonary function indices. (S1 Table) Due to the complexity of cardio-pulmonary interplay, the association between cardiac performance and ventilation abnormalities did not always imply causation.

### Prognostics impacts of pulmonary functions in HFpEF

In the Norwegian Heart Failure Registry of 4,132 HF patients, COPD was independently associated with a 19% excessive risk of mortality during a mean follow-up duration of 13.3 months [32]. In addition, Andrea et al. further suggested that impaired pulmonary function was predictive of long-term mortality in a relative small population of 71 HFpEF patients [33]. While obstructive ventilation, rather than restrictive airflow pattern, was correlated with long-term survival in patients with HFrEF [34], Andrea et al. also proposed that the presence of airflow limitation was a major prognostic factor for mortality and cardiovascular hospitalization in patients with HFpEF [33].

In this study, we reported similar findings that COPD was a risk factor of all-cause mortality in Asian patients with HFpEF. Both obstructive and restrictive ventilatory indices were independently associated with long-term outcomes in patients without COPD. However, only the small airway function, as indexed by FEF25-75, was independently predictive of mortality in those without COPD. Because the significance of FEF25-75 was marginal (p value = 0.044), it was possible that the results were due to chance alone. However, given that both FEV1 and FEF25-75 were reduced in COPD patients, the study result might support FEF25-75 as a more sensitive marker for small airway function. Our novel findings suggest distinct pathophysiology in patients with and without COPD, reflecting how cardiac performance impacts the pulmonary functions.

### Study limitations

There were several study limitations in this work. Most of the HF population in this registry was composed of elderly subjects, therefore HFpEF was prevalent. Selection bias arising from the unobserved variables might have been present. However, we have adjusted for the available confounders to evaluate the independent prognostic values of pulmonary function in patients with HFpEF. Although neither NT-proBNP nor BNP was available in the study, the diagnosis of COPD and HF was conducted by the clinicians based on the echocardiographic and pulmonary function examinations. While body plethysmograph was only available in 818 patients, we may not have sufficient power to conclude that predicted %TLC was not related to mortality in multivariate Cox proportional hazard model. However, subjects with or without body plethysmograph shared similar risks for mortality. Also, we quantitate small airway function by FEF25-75, not by nitrogen washout test or impulse oscillometry. Lastly, data of HF re-hospitalization was not available in the present study. Further work was needed to address the associations for mortality and morbidity.

## Conclusion

In the ambulatory patients with HFpEF, 27.6% of them had concomitant COPD. Subjects with COPD had poorer long-term survival than those without COPD. For patients without COPD, ventilatory abnormalities were associated with cardiac performance and predictive of long-term survival. Among patients with COPD, only FEF25-75 was associated with clinical outcomes. The results may suggest the ventilatory abnormalities prevails in subjects with HFpEF, regardless of COPD, and it is related to long-term outcomes. The present study may support the need for comprehensive pulmonary function tests in patients with HFpEF for clinical risk stratifications.

## Supporting information

S1 Table Multivariate linear regression analysis to determine the independent predictors of pulmonary function parameters in HFpEF subjects without COPD. (DOCX)
