# Supplementary material for: The role of pulmonary function in patients with heart failure and preserved ejection fraction: Looking beyond chronic obstructive pulmonary disease
Source: PLoS One. 2020 Jul 7;15(7):e0235152. doi: 10.1371/journal.pone.0235152 (PMC7340281; doi:10.1371/journal.pone.0235152)
Supplement: S1 Table — (DOCX) [file pone.0235152.s001.docx]

| **Supplement Table 1. Multivariate linear regression analysis to determine the independent predictors* of pulmonary function parameters in HFpEF subjects without COPD** | | | |
| --- | --- | --- | --- |
|  | **Partial R-squared** | **Standardized coefficients** | **P value** |
| **VC% predicted (Model R-squared=0.108)** | | | |
| **PASP** | 0.108 | -0.331 | <0.001 |
| **FEV1% predicted (Model R-squared=0.092)** | | | |
| **PASP** | 0.092 | -0.303 | <0.001 |
| **FVC% predicted (Model R-squared=0.107)** | | | |
| **PASP** | 0.101 | -0.287 | <0.001 |
| **Septal E/E’** | 0.006 | -0.092 | 0.016 |
| **RV to TLC ratio (Model R-squared=0.076)** | | | |
| **PASP** | 0.055 | 0.211 | <0.001 |
| **Septal E/E’** | 0.007 | 0.131 | 0.009 |
| **LV mass** | 0.008 | -0.104 | 0.029 |
| **TLC% predicted (Model R-squared=0.071)** | | | |
| **PASP** | 0.064 | -0.239 | <0.001 |
| **LV mass** | 0.007 | -0.095 | 0.038 |
| ***stepwise adjusted septal E/E’, PASP, LA diameter, and Left ventricular mass**  FEF 25 to 75%: forced expiratory flow at 25-75% of the pulmonary volume, FEV1: forced expiratory volume in 1st second, FVC: forced vital capacity, LA diameter: the diameter of left atrium, LV mass: left ventricular mass, PASP: pulmonary artery systolic pressure, RV: residual volume, TLC: total lung capacity, VC: vital capacity | | | |
